# The *in-related self*: reclaiming *Paarung* in critical phenomenological psychopathology

**DOI:** 10.3389/fpsyg.2025.1602106

**Published:** 2025-08-07

**Authors:** Elena Billwiller

**Affiliations:** Università degli Studi di Padova, Padua, Italy

**Keywords:** phenomenological psychopathology, critical phenomenology, feminist phenomenology, embodied subjectivity, racialized and gendered experience, *Paarung* (passive synthesis), relational individuation

## Abstract

This article explores the conceptual and clinical implications of integrating phenomenological psychopathology with critical and feminist phenomenology. Drawing on the Husserlian concept of *Paarung* – understood as a passive, embodied synthesis grounding the constitution of the other – we develop a framework for interpreting perceptual disruptions in subjects affected by social oppression. After outlining the methodological foundations of phenomenological psychopathology, we show how critical approaches expand this tradition by foregrounding the socio-historical structures that shape embodied experience. To articulate the effects of power on perceptual life, we introduce the notion of *malign Paarung*, which designates the pathological sedimentation of social norms into embodied relationality, producing alienation and inhibiting reciprocity. The analysis focuses on two emblematic configurations: temporal disruption in racialized subjectivities (Fanon, Al-Saji) and spatial inhibition in gendered embodiment (Young, Sullivan). These are not fixed associations, but heuristic articulations aimed at clarifying how different structures of domination distort the temporal and spatial dimensions of experience in interwoven ways. The final section argues for a therapeutic appropriation of Paarung within the clinical encounter, conceived not as a neutral act of diagnosis but as a co-constitutive process capable of reorganizing disrupted experiential structures. Within this framework, relational individuation (*in-related self*) emerges as both an epistemological and ethical horizon of care, oriented toward the co-emergence of shared meaning and emancipatory forms of subjectivity.

## 1 Introduction to phenomenological psychopathology

Exactly seventy years have passed since J. H. Van den Berg’s preface to “The Phenomenological Approach to Psychiatry” (1955), where he described phenomenology as a still-young discipline, characterized by a limited body of work, often highly theoretical and difficult to read. In reality, even at that time, phenomenology had already gained considerable traction within both philosophical and psychiatric contexts, thanks to contributions by Jaspers, Binswanger, Minkowski, and others. What Van den Berg sought to emphasize was not the theoretical development of phenomenology itself, but rather the limited systematization of its clinical application within the Anglophone psychiatric world.

Despite these historical clarifications, one element of Van den Berg’s perspective remains compelling today: the idea – already central in his work – of phenomenology as an attitude faithful to the things themselves (Van den Berg, 1955). That is, a way of observing that is valid not only for science and psychology but that – as the author insists – “is not at all new for human beings in general” (Van den Berg, 1961, p. 70).

This conception directly connects to the method of Daseinsanalyse (or Daseinsanalytik), understood as a phenomenological anthropology applied to psychiatric knowledge and developed by Ludwig Binswanger as an authentic “science of the human being.” Not by chance, Van den Berg dedicated his book to Binswanger, whom he rightly regarded as the father of phenomenological psychiatry (Binswanger, 1922, 1942, 1946, 1951).

It is, however, important to distinguish Binswanger’s clinical contribution from that of Karl Jaspers, who is generally acknowledged as the founder of phenomenological psychopathology due to his “General Psychopathology” (1913), which introduced the methodological distinction between “explanation” (*Erklären*) and “understanding” (*Verstehen*).

A detailed reconstruction of the development of phenomenology during the period in which phenomenological psychopathology took shape – and its intersections with contemporary psychology – falls outside the scope of this article and would require a separate inquiry. However, it is essential to recall Dilthey’s (1924) reflections on the nature of psychological method, where he famously states: “we explain nature, but we understand psychic life” (Die Natur erklären wir, das Seelenleben aber verstehen wir).

This principle was later taken up and systematized by Jaspers in his book (1913), where the distinction between *Erklären* and *Verstehen* is explicitly articulated. According to Jaspers, while the natural sciences rely on explanatory methods based on causal and objective analysis, psychopathology requires a hermeneutic method oriented toward grasping the internal meaning and subjective structure of lived experience. This methodological shift marks the emergence of phenomenological psychopathology as an autonomous discipline, grounded in attentive listening and rigorous description of subjective experience. As Van den Berg emphasized, the aim is not simply to explain symptoms, but rather “to offer the most complete and accurate description possible of what the healthy or the ill person experiences” (Van den Berg, 1961, p. 101).

If the object of inquiry in psychology and psychopathology is the human being in their entirety, the phenomenological approach demands that we move beyond the diagnostic classification of isolated psychic elements to grasp the patient’s lived world as a whole. In this perspective, Binswanger, in defining psychiatry as a “science of the human being,” writes:

“[With] the title of my talk, *Psychiatry as a Science of the Human Being*, I mean from the outset to indicate that the ground and foundation upon which psychiatry as an autonomous science may take root is neither the anatomy and physiology of the brain, nor biology, nor psychology, characterology, or typology in general, nor even the science of the person—but the human being” (Binswanger, 2013, p. 37).

From this standpoint, it is important to note that psychic trauma is not understood as an objective entity or an isolable clinical fact, but rather as a rupture in the subject’s situated existence – a crisis in their relation to the world. Van den Berg insists on this point as well, writing that “the situation makes psychic trauma possible, or creates it” (Van den Berg, 1961, p. 87). This formulation underscores that a traumatic event cannot be understood apart from the relational and symbolic context that renders it meaningful. Trauma, then, does not exist in itself, but is constituted phenomenologically within a shared structure of meaning.

Consequently, for the phenomenological psychiatrist, it is essential to attend not only to the symptom or diagnosis, but to the situation in its full complexity: to the lived world that has become real for the patient. Therapeutic intervention cannot be reduced to etiological inquiry or nosographic classification – it requires a direct involvement in the patient’s existence. In this light, the clinician is not an external observer, but a *Daseinspartner*, a companion in the existential practice of care.

“The phenomenologist seeks to grasp the physiognomy of things as the patient perceives them; to put it more simply, the aim is to understand their existence in depth, before venturing any judgment about it. […] Phenomenological psychiatry does not claim to offer new therapeutic methods; rather, it expresses in new words what has always been the foundationof this most human of vocations: healing the patient through word and action” (Van den Berg, 1961, p. 115).

This perspective restores to psychiatric practice its genuinely human dimension, grounded in intersubjective encounter and the shared inhabitation of the patient’s existence. As Binswanger writes:

“We can only understand madness on the ground [*Grund*] of our common human condition – on the basis of the *conditio humaine* – or, which is the same, if we recognize even in the mad person another human being [*Mit-Mensch*]” (Binswanger, 2013, p. 39).

The clinician’s phenomenological gaze must therefore extend beyond the patient’s immediate lived world to encompass the body, time, space, and personal past – all elements that structure existence. It is through the contribution of phenomenology that these dimensions have entered fully into psychopathological analysis (Binswanger, 1973, 2022; Minkowski, 1968), surpassing the positivist approach that had long deemed them irrelevant.

To speak of body, space, and time is to refer to fundamental categories of experience, present in every subjectivity. However, although these structures are constitutive of lived experience, their manifestation is neither fixed nor universal: it varies according to the cultural, sociopolitical, and power relations that shape the subject’s mode of inhabiting the world. In this regard, Van den Berg’s remark (1961) remains particularly relevant: psychic trauma is not an isolated datum, but must be understood in relation to the context and situation in which it emerges.

It becomes thus essential to revisit the concept of “being-in-the-world” (In-der-Welt-sein), as elaborated by Binswanger (2018), according to which human existence is intrinsically relational and structured by a bi-directional dynamic between the subject and the world. The individual is never a detached entity, but is constitutively embedded in a network of relationships that shape experience. Subjective experience is therefore not the exclusive product of internal processes, but is co-constituted through the subject’s relation to their environment and to others.

Building on these premises, phenomenological psychopathology has the merit of restoring centrality to the patient’s lived experience and situated existence. However, it is now essential to ask how this approach might be expanded to include the power structures and social contexts that profoundly shape subjectivity. In this direction, the encounter with critical and feminist phenomenology becomes a pivotal step: these frameworks allow us to problematize the apparent neutrality of fundamental structures of perception – body, space, and time – by showing how they are historically and socially situated.

This article aims to explore whether, and how, phenomenological psychopathology can be reoriented through a critical lens capable of integrating into clinical practice the perceptual transformations induced by racialization, sexism, and other forms of oppression. This attempt aligns with the renewed vitality of phenomenology in contemporary psychiatry, as attested by recent works such as “The Oxford Handbook of Phenomenological Psychopathology” (Stanghellini et al., 2019), and is further developed in dialog with theoretical proposals addressing the epistemological and political status of subjectivity, such as “Critical Phenomenology and Psychiatry” (Zahavi and Loidolt, 2022).

The guiding hypothesis of this inquiry is that subjectivity does not form in isolation, but through a network of bodily, perceptual, and symbolic relations that may be disrupted, distorted, or denied in oppressive contexts.

The article is structured in six sections. Following this introduction (Chapter 1), Chapter 2 reconstructs the contribution of critical and feminist phenomenology, with particular attention to the role of social structures in the constitution of the self. Chapter 3 proposes a reinterpretation of the Husserlian concept of *Paarung* as a primary relational structure and explores its relevance for understanding perceptual disturbances in clinical contexts. The following chapters (4 and 5) analyze the alterations of temporality and lived space in contexts of racialization and sexism. The sixth and final chapter discusses the clinical and ethical implications of a critical phenomenological approach, showing how the therapeutic relationship may function as a space for the co-constitution of meaning and the reconfiguration of experience.

## 2 The contribution of critical and feminist phenomenology

As we have seen, phenomenological psychopathology marked a significant shift away from positivist psychiatry by refocusing attention on the complexity of subjectivity and the analysis of lived experience. Drawing on and reworking the methodological insights of Husserl, Heidegger, and existential phenomenology more broadly, it opened the way for an approach more attuned to the patient’s world. This article proposes a critical extension of that tradition by exploring the possibility – and urgency – of integrating into phenomenological psychopathology the tools offered by critical and feminist phenomenology (Fisher, 2000; Weiss et al., 2020). The aim is to expand the understanding of subjectivity by incorporating socio-cultural, racial, and gendered dimensions – an aspect not yet systematically addressed by classical phenomenology.

Over the past few decades, critical phenomenology has developed a systematic reflection on how power structures – internalized and sedimented in both individual and collective behavior – profoundly shape how subjects perceive themselves and relate to the world. It reveals that subjectivity is not a matter of pure interiority, but is always formed within historical and normative contexts that condition its expressive possibilities.

“In this context, critical phenomenology seeks to highlight how the self is not conditioned by power-relations as a passive medium, but also how the boundaries between self, other, and social norms are often liminal spaces that allow degrees of ambiguity” (Magrì and McQueen, 2023, p. 22).

Far from rejecting the phenomenological method, the critical approach renews it by interrogating the material, affective, and historical conditions that structure experience. In this sense, it operates at the intersection of feminist philosophy, critical race theory, and contemporary research on embodiment, illness, and sociality:

“Critical phenomenology cannot be neatly separated from feminist philosophy, critical race theory, and contemporary phenomenological research on perception, embodiment, illness, and sociality” (Magrì and McQueen, 2023, p. 23).

Through its dialog with these fields – including Disability Studies and the Medical Humanities (Carel, 2016) – critical phenomenology adopts an intersectional perspective capable of investigating how gender, race, ability, and power intersect in the constitution of identity and lived experience. While classical phenomenology has contributed to the understanding of space and time as foundational categories of experience, critical phenomenology invites us to question how these very categories are shaped by the power relations that structure everyday life and the ways in which subjects inhabit the world.

Integrating critical phenomenology into psychopathological practice means recognizing that social inequalities – such as those rooted in experiences of sexism, racism, or ableism (McRuer, 2006) – not only affect the constitution of the self, but may also contribute to the emergence of perceptual disturbances and pathological experiences. If, as Van den Berg (1961) showed, phenomenological psychopathology highlights the situated nature of experience, it becomes crucial today to ask which subjects are able to experience time as an open and linear progression, and which are instead compelled to live under conditions of suspension, arrest, or forced iteration. Who enjoys the freedom to move through space, and who, by contrast, is constrained by spatial and bodily restrictions imposed by normative oppression?

Such questions lie at the core of critical-phenomenological inquiry. While remaining faithful to the phenomenological method, this approach concentrates on the structural conditions that make experience possible – or limit it. From this standpoint, space, time, and the body are not merely transcendental categories of subjectivity, but domains traversed by asymmetries of power that profoundly shape perception and lived experience. The forms of alienation that stem from such inequalities affect the subject’s capacity to orient themselves in the world, to generate shared meaning, and to access stable forms of self-awareness.

The integration of critical phenomenology thus enables an expansion of the scope of phenomenological psychopathology, offering new tools for understanding the variety of subjective experience. It invites us to consider that psychological suffering is not merely a deviation from clinical norms, but must also be analyzed in terms of the material and symbolic conditions that shape human lives. In this way, phenomenological analysis does not stop at describing the intentional structures of consciousness, but opens up to a broader understanding of subjectivity as always already socially, affectively, and historically situated.

If space, time, and the body are fundamental categories of lived experience, critical phenomenology urges us to see how they are also traversed by power relations and embodied histories. The body, in particular, is never neutral: it is a situated body, shaped by social and cultural norms that regulate visibility, freedom of movement, and expressive possibility. In this light, Alcoff’s (2006) concept of “visible identities” shows how social identity is not a fixed and individual structure, but a relational and contextual process – one that may become either a constraint or a resource, depending on one’s position within power relations.

The body is thus not simply a biological organism, but a point of intersection between subjectivity and social norms. Sartre’s (2012) phenomenology of the *body-for-others* reveals how the lived body is continually exposed to the gaze of others and is therefore vulnerable to categorization, shame, and alienation. Related to this is Merleau-Ponty’s (2003) notion of the *habitual body*, according to which habit constitutes a sedimented perceptual and affective structure through which social norms and expectations are incorporated.

This perspective has been developed by authors such as Ngo (2017) and Sullivan (2001), who demonstrate how many bodily postures and perceptual modalities are racialized or sexually coded, and contribute to the reproduction of discriminatory dispositions. In dialog with Bourdieu’s theory of *habitus* (1977), these investigations highlight how bodily experience is deeply socialized and reflects unequal structures of possibility and access to agency. The concept of *ableism*, as theorized by McRuer (2006), also fits within this framework, showing how the ideal of the healthy, efficient, and autonomous body functions as an exclusionary norm – one that marginalizes experiences of disability and vulnerability.

Building on these references, it becomes evident that identity cannot be understood as an internal, stable, or original property, but rather as a process continuously negotiated in relation to the other. This insight brings us back to the heart of Husserlian phenomenology and, in particular, to the concept of Paarung, which will be the focus of the next section. As we shall see, Paarung refers to a fundamental structure of perceptual pairing between self and other, which makes possible the recognition of the other as a subject similar to oneself. We propose to rethink this structure in light of the critical and feminist reflections outlined above, in order to examine how Paarung may be hindered, distorted, or denied within contexts of racialization, sexualization, or marginalization – thereby compromising the intersubjective foundations of perception and opening new pathways for understanding spatial and temporal disturbances in clinical settings.

## 3 The concept of *Paarung* as relational identity

Critical and feminist phenomenology have shown how power relations profoundly shape lived experience, modulating one’s perception of the body, temporality, and spatiality. However, to understand how relational identity is constituted – that is, the experience of the other as “another like me” – it is necessary to return to a more originary level of subjectivity. In this context, the Husserlian concept of *Paarung* (pairing) assumes a central role.

In “Analyses Concerning Passive Synthesis” (Hua XI), Husserl (2001) introduces Paarung as a form of passive synthesis: a pre-reflective experience in which two similar appearances – such as two lived bodies – are associated without requiring any explicit intentional act. This elementary mechanism enables the constitution of the other as an *alter ego*, that is, as a subject endowed with an interiority analogous to one’s own. As Husserl writes:

“Every Paarung is a passive synthesis, an original experience that unites two corresponding appearances and allows us to constitute the experience of a foreign I as a lived body” [Jede Paarung ist eine passive Synthesis, sie ist ein ursprüngliches Erlebnis des Zusammenschlusses zweier einander entsprechenden Erscheinungen, das uns die Erfahrung eines fremden Ich als Leib konstituiert,” Husserl, *Analysen zur passiven Synthesis* (Hua XI), p. 135].

*Paarung* is thus the originary mechanism of embodied intersubjectivity: it enables consciousness to constitute the other not as an object, but as a lived body (*Leib*), and therefore as a subject. This is not an abstract or intellectual resemblance, but an analogical perception grounded in “indirect intentionality” (Besoli, 2018), rooted in bodily manifestations and sensory proximity.

The concept of Paarung, however, is not confined to the “Analyses Concerning Passive Synthesis”; it recurs throughout Husserl’s oeuvre, including in key texts such as the “Logical Investigations,” the “Cartesian Meditations” (especially §51) (Husserl, 2020), “Experience and Judgment,” and the lectures on genetic phenomenology (Husserl 1982). Its significance lies in the double structural function it performs: on the one hand, it provides the genetic basis of intersubjectivity, making the experience of the other possible; on the other, it operates as a universal mode of constitution, playing a decisive role in the genesis of subjectivity and in the construction of a shared world.

If, in Husserl’s genetic phenomenology, Paarung represents the originary moment of alterity’s constitution, more recent scholarship has emphasized its potential as an embodied relational principle capable of illuminating the concrete genesis of situated subjectivity (Steinbock, 1995; Zahavi and Loidolt, 2022; Doyon, 2021).

From this perspective, Paarung no longer merely functions as a passive structure; it becomes a key theoretical tool for understanding intercorporeal reciprocity and the socio-historical embeddedness of subjectivity. As Besoli (2018) has clarified, Paarung does not occur on a purely spiritual or intellectual level, but emerges from a concrete experience guided by an indirect intentionality grounded in embodied subjectivity. The lived body (*Leib*) is the very condition of possibility for encounter: only through bodily proximity can we recognize the other as a subject, rather than merely an object in the world.

This recognition, however, does not imply fusion or identification; rather, it preserves an irreducible distance that grounds intersubjectivity as a structurally differential relation. On this reading, the *I* is not constituted in isolation, but always already in relation to another, within a historically situated coexistence. Subjectivity is not a closed transcendental monad; it is, from the outset, open to a horizon of reciprocity: what Husserl describes as the *apperception* of the other is made possible by a shared belonging to an intercorporeal and intersubjective lifeworld. This co-belonging is never neutral, but is structured through time, history, and the concrete sociality of subjects: every *I* is also a *socius* (Besoli, 2018; Zahavi and Loidolt, 2022).

It is precisely this structural openness to the other that may undergo fundamental distortions. Experiences of racialization, sexism, ableism, or social marginalization can profoundly disrupt the process of Paarung, hindering the activation of empathetic recognition and producing pathological forms of relationality. As shown by Fanon (2015) and Young (2005), the oppressed body becomes a disoriented and disaligned body – one that can no longer rely on the environment or others as sources of shared meaning.

From being a genetic structure of intersubjective constitution, Paarung can thus be reinterpreted – through a critical and feminist lens – as a clinical indicator of the quality of embodied relationality. In psychopathological disorders, in particular, its distortion manifests in multiple forms: in the loss of temporal synchronization (as in depression), the disintegration of body schema (as in dissociative disorders), or the fragmentation of lived spatiality (as in post-traumatic disorders) (Fuchs, 2013; Ratcliffe, 2015). In each of these cases, what is compromised is the capacity to enter into resonance with the other – a resonance that, as the concept of Paarung shows, is not secondary, but rather a structural foundation of subjectivity.

The critical reinterpretation of Husserl’s concept of Paarung shifts the focus from a strictly transcendental level to one that is embodied and situated, demonstrating that subjective identity emerges from a primary relational process that is structurally vulnerable to disruption. If Paarung constitutes the original mechanism through which the other is recognized as *like me* and thus rendered accessible to experience, then it becomes clear that the interruption of this dynamic affects the very capacity of the subject to orient herself in the world, to feel part of a shared horizon, and to construct a stable sense of identity.

From this perspective, understanding psychopathological suffering requires inquiry not only into the subject’s internal alterations, but also into the relational, historical, and symbolic conditions that undermine the very possibility of Paarung. Disruptions in embodied pairing do not occur in abstraction; they arise within specific cultural and political constellations that radically shape the temporality, spatiality, and corporeality of lived experience.

It is precisely here that clinical phenomenology and critical phenomenology can fruitfully converge: in the attempt to understand how forms of psychological suffering may arise from systemic failures in the embodied recognition of the other.

To think Paarung in therapeutic terms thus means recognizing that the clinical relationship can become a space for perceptual and intersubjective reactivation. The therapist, through an attitude of embodied, empathetic, and historically informed openness, can help reestablish the minimal conditions for embodied reciprocity, offering the patient the often-lost experience of being seen, heard, and recognized as a subject. In this sense, Paarung is not only an epistemological concept but also a clinical and ethical stake at the core of the therapeutic process.

From these premises, the following sections will examine two paradigmatic cases in which Paarung is profoundly disrupted in its embodied and relational foundations. The first, through the reflections of Frantz Fanon, will show how the experience of time is distorted in the lived experience of racialized subjectivity; the second, drawing on the analysis of Iris Marion Young, will focus on the spatial transformations that affect female bodies within a society structured by sexist norms. In both cases, phenomenological clinical practice may find new resources by critically interrogating the conditions that prevent a symmetrical relation between self and other.

## 4 Temporal alterations

Temporality is not a universal and invariant phenomenological structure; rather, it is constituted through the embodied relation to the world, shaped by historical, social, and symbolic mediations. In contexts of racialization, this constitution becomes distorted: lived time appears fragmented, discontinuities between past, present, and future intensify, and the existential openness toward the future is disrupted. Frantz Fanon was among the first to thematize this temporal fracture in the experience of racialized subjects, describing colonial experience as a suspension of historical time and as a compression of existence within a “frozen space–time” (Fanon, 2015). Building on this analysis, Alia Al-Saji has shown how racialization directly affects embodied temporality, producing aberrations in affect, agency, and experiential rhythm – phenomena that cannot be reduced to individual disorders but rather reflect an interiorized social pathology (Al-Saji, 2013).

This section thus aims to analyze the structural link between race and time, interrogating how racialized subjectivities experience temporality under conditions of historical alienation and systemic oppression. To this end, we will articulate Husserl’s concept of Paarung – the passive associative synthesis underpinning the constitution of alterity – with the notion of *malign Paarung* (Kitwood, 1997), understood as a pathological form of perceptual and embodied association that prevents the emergence of a symmetrical relation to the other and to one’s own futurity.

In the introduction to “Black Skin, White Masks,” Fanon writes:

“The architecture of this work is rooted in the temporal. Every human problem must be considered from the standpoint of time. Ideally, the present will always contribute to the building of the future. And this future is not the future of the cosmos but rather the future of my century, my country, my existence” (Fanon, 2015, p. 14).

This passage highlights how time is not lived uniformly but is profoundly affected by the historical and social conditions in which the subject is embedded. Fanon analyzes how racialization distorts temporal experience, imposing a rupture between the present and the past of colonized subjects, and rendering the future into an uncertain and alienated horizon.

Alia Al-Saji, one of the leading figures in critical phenomenology, develops Fanon’s insights in her article “Too Late,” where she explores the phenomenology of racialized temporality:

“Attending to the temporal dimensions of racialization raises the problem of method. […] If racism is reflected not only in economic, social, and political conditions, but also structures lived experience, then anomalies and breakdowns in experience cannot be studied as purely individual afflictions in racial societies. The study of the ways in which racism is lived – of the ‘aberrations of affect’, embodiment, agency, and temporality that accompany it – raises the question of how psychopathology may crystallize social pathology, and how phenomenological method can do justice to racialized experience” (Al-Saji, 2013).

This perspective reveals how racialization is not merely an external or socioeconomic phenomenon, but penetrates lived experience, shaping temporal perception and generating anomalies that cannot be reduced to individual pathologies.

From a phenomenological standpoint, this experiential blockage may be understood as a distortion of Paarung: instead of operating as an embodied recognition of the other as “like me,” passive association becomes fixated on imposed social images, projected onto the Black body as a bearer of radical alterity. The other, in this case, is not appresented in their subjectivity but reduced to a stigmatizing image that forecloses any possibility of reciprocity.

We propose to refer to this pathological form of association as *malign Paarung*, drawing on an insight by Kitwood (1997), who argues that identity may become deformed when encounters with others are structured by negative expectations and stereotypes. Within racialized contexts, *malign Paarung* manifests as a dysfunctional perceptual coupling that obstructs the constitution of the other as a subject and binds the self to an alienated temporality.

In phenomenological terms, this results in a collapse of temporal intentionality: the flow of experience cannot unfold freely, as it is anchored to a closed and imposed past. The lived temporality of the racialized subject loses its projective openness, becoming a compulsion to repeat: every gesture, every utterance, every presence is interpreted through a sedimented historical schema that forecloses the novelty of the encounter. Paarung, which normally sustains the continuity of intersubjective world-constitution, becomes rigidified into a repetitive and pathological form.

Integrating the concept of Paarung into phenomenological psychopathology can offer valuable insights in this regard. If racialization generates dysfunctional passive associations between past and present, therapeutic intervention may aim to interrupt these pathological linkages and facilitate a spatiotemporal reorganization of the patient’s lived experience. In clinical practice, this means working not only on the narrative reconstruction of the self, but also on the possibility of reinscribing the past within a renewed temporal configuration – one that allows the subject to access a future not wholly determined by internalized oppressive images. This reflection on temporal distortions is directly connected to the analysis of spatial and bodily alterations, which represent the other fundamental dimension of experience under conditions of oppression.

The legacy of Frantz Fanon finds further development in the works of Johnson (1993) and Yancy (2008), both of whom deepen the understanding of how racialization shapes the embodied experience of oppressed subjects. Johnson, building on Fanon’s notion of the “epidermal schema,” introduces the concept of “epidermalization” to describe how skin color becomes a social marker that conditions self-perception and intersubjective recognition. Yancy, through his analysis of the “structural hegemonic order,” emphasizes the performative power of what he terms the “white gaze,” showing how it objectifies and reduces the racialized body to a visible entity, unrecognized in its full subjectivity. In a striking passage, Fanon recounts:

“In the white world the man of color encounters difficulties in the development of his bodily schema. […] I discovered my Blackness, my ethnic characteristics; and I was battered down by tom-toms, cannibalism, intellectual deficiency, fetishism, racial defects, slave-ships, and above all: yes, above all, the grinning Y a bon Banania” (Fanon, 2015, p. 112).

These processes are central to critical phenomenology, which reveals that racialization is not only a political or social fact, but also a distorted perceptual experience that undermines the integrity of the self. Racialized subjects undergo a fracture in temporality, remaining anchored to a past that repeats itself and restricts autonomous projection into the future. At the same time, this alienation manifests spatially through mechanisms of invisibilization and de-subjectification that render the racialized body a mere object of perception – rather than a subject acting in and through the world.

Olkowski (2021) has emphasized that racism operates through a form of “paradoxical temporal duality,” one that represses the actual memory of colonized peoples and replaces it with stereotypes and historical distortions, thereby legitimizing colonial domination in both the present and the future. Racialized temporality is thus an interrupted temporality, which imposes on subjects a sense of perpetual delay (“lateness”) and a field of possibilities already preempted by the dominant culture (Al-Saji, 2013; Olkowski, 2021). This analysis also applies to other forms of oppression – such as sexism and social marginalization – which impose a systemic reduction of agency and futurity upon historically subordinated subjects.

Finally, to fully grasp the distortion of Paarung in racialized and gendered contexts, it is necessary to consider the early origins of the discriminatory gaze. Following a critical-phenomenological approach, one can argue that racist and sexist perception is not merely the outcome of explicit beliefs, but emerges through early affective and embodied socialization. As Bourdieu suggests, such structures become embedded in perceptual *habitus*, sedimented through childhood trajectories and reproduced in gestures, affects, and patterns of visibility. This confirms the evolutionary and pre-reflective roots of discriminatory dispositions, extending far beyond the domain of explicit cognition. In this direction, Alia Al-Saji demonstrates how racialized temporality operates already at the level of perception, structuring the encounter with the other as an asymmetrical relation, prefigured by stereotypes and implicitly internalized expectations.

These temporal and spatial perceptual distortions are deeply intertwined: if the lived time of oppressed subjects is fragmented and trapped in a cycle imposed from the outside, space is likewise regulated by power structures that determine who may move freely and who is subject to spatial and bodily constraints. The next section will explore this dimension through the lens of feminist phenomenology, which has foregrounded the role of gender and embodiment in shaping oppression and the lived structure of experience.

## 5 Spatial and corporeal alterations

The reflection on temporal perceptual disruptions linked to the racialized experience of time finds a natural continuation in the analysis of spatial and corporeal alterations that shape gendered subjectivity in patriarchal contexts. Just as the temporality of oppressed subjects may appear fragmented and suspended, so too the lived space and the body inhabiting it are profoundly shaped by normative and relational constraints that configure self-perception in accordance with prevailing power structures.

In this regard, feminist phenomenology has provided crucial analytical tools. In particular, Iris Marion Young’s essay “Throwing Like a Girl” (2005) stands as a foundational reference for understanding how the feminine body is lived and internalized under conditions of sexist oppression. Young shows how gender socialization trains the feminine subject to hold back, to limit motor initiative, as if anticipating judgment or the threat of failure. As she writes:

“The modalities of feminine bodily comportment, motility, and spatiality that I have described here are, I claim, common to the existence of women in contemporary society to one degree or another. They have their source, however, in neither anatomy nor physiology, and certainly not in a mysterious feminine essence. Rather, they have their source in the particular situation of women as conditioned by their sexist oppression in contemporary society” (Young, 2005, p. 32).

A key concept is that of *inhibited intentionality*, which Young uses to describe the pre-reflective mode by which the body withdraws, refrains from fully acting, and is experienced as fragile and under observation:

“Inhibited intentionality describes the experience in which the body’s capacity for movement and action is available, but the subject hesitates to enact it, or enacts it only partially, because the world is not experienced as open to her agency” (Young, 2005, pp. 36–37).

From a phenomenological standpoint, the experience of space is relationally structured and affectively inhabited – an expression of a passive coupling between the feminine body and a normative system that defines not only where one is legitimately allowed to be, but also how one may move, express oneself, and appear.

In contexts shaped by oppressive power relations, this perceptual coupling – far from generating reciprocity – tends to crystallize into pathological forms, marked by alienation, control, and inhibition. This is what we have previously described as *malign Paarung* (Kitwood, 1997): a relational pairing that embodies dominant norms to the point of severely restricting embodied agency.

The consequences of this perceptual distortion sediment early in the body in the form of motor, affective, and postural patterns, structuring what Bourdieu has defined as *habitus* (Bourdieu, 1977): embodied dispositions that regulate how the body inhabits space and becomes available to others. As with the racist gaze, the sexist gaze is also rooted in an implicit pedagogy that assigns feminine bodies a contained, secondary, and hypervisible position, instituting from the outset a compulsion toward spatial performativity.

Such spatial distortions may take the form of forced invisibility, but more insidiously, they often appear as a constant demand for emotional presence and relational availability. Sullivan (2006) has proposed the concept of *ontological expansiveness* to describe the habitual comportment of privileged subjects – white, male, able-bodied – who move through the world as if all space were rightfully theirs, expecting others to continuously modulate their affect and movements so as not to disturb their existential comfort.

This dynamic entails significant psycho-corporeal costs for racialized and gendered subjectivities, who are forced to manage the emotional landscape of others at the expense of their own agency and self-care. It constitutes a pervasive form of relational *habitus* inscribed in the body – producing structural microaggressions for the subordinated and normalized microprivileges for the dominant – thus contributing to the reproduction of spatial and affective hierarchies through everyday gestures, postures, and motor habits.

Critical and feminist phenomenology thus offers conceptual tools for interpreting these forms of distress not as individual pathologies, but as expressions of embodied power relations that manifest in the spatiality of the body and its exposure to the gaze of others. If Paarung is the structure that enables the constitution of relational identity through the embodied experience of the other, then its distortion in sexist contexts directly challenges clinical practice.

It is on this ground that the possibility for a therapeutic reflection emerges. In the next section, we will explore how the concept of Paarung may be integrated into the clinician-patient relationship – not only to grasp spatio-temporal perceptual disturbances, but also to envision new forms of co-constructed experience and rehabilitation of the self as a relational subject.

## 6 Clinical relationship and ethical implications: *Paarung* as therapeutic co-constitution

The integration of phenomenological psychopathology with critical and feminist phenomenology entails not merely a thematic broadening of the analysis, but a reconfiguration of its methodological foundations. While phenomenological psychopathology – starting with Jaspers (1913) and Binswanger (1946) – emphasized the importance of the *epoché* and the intentional analysis of experience, contemporary critical approaches suggest moving beyond the isolated analysis of consciousness to interrogate the silent operations of historical and social structures in its formation. In this regard, critical phenomenology proposes a *critical suspension* (Al-Saji, 2010), one that suspends not only subjective judgments but also the normative frameworks sedimented within clinical and social perception – frameworks that shape what is experienced as “normal,” “pathological,” or even “visible.”

This methodological shift requires a rethinking of the clinician’s stance. Critical phenomenology reveals that every therapeutic encounter is situated within power relations that condition not only how the patient is seen and heard, but also how they perceive their own body and history (Weiss, 2016; Carel, 2016). The Husserlian concept of Paarung, already described as an associative principle and originary relational structure, can be redeployed in this context as an epistemological guide. It allows us to reconceive the clinical relationship not as a meeting between isolated subjects, but as a co-constitutive process through which self and other are mutually articulated within shared spatio-temporal configurations.

This perspective shifts the aim of clinical work beyond diagnostic understanding of symptoms, toward a reorganization of lived experience by reactivating possibilities that have been interrupted or denied. The therapeutic relationship becomes a space of perceptual resonance and disruption of the passive associative chains that fuel alienation. In this sense, the critical-phenomenological method takes shape as a situated hermeneutics of lived experience – capable of both recognizing the social structures sedimented in the patient’s body and temporality, and opening up space for rearticulating the self through a new, therapeutic Paarung with the world.

Within the clinical encounter, Paarung must not be reduced to mere empathic attunement or intuitive identification of symptoms. Rather, it constitutes an embodied structure of co-constitution, capable of moving beyond the traditional diagnostic paradigm – centered on classification and objectification – toward a phenomenological framework that foregrounds the subject’s individuation in their processual and historical unfolding.

Whereas identification tends to fix identity through the reiteration of predetermined categories, individuation refers to a dynamic, relational, and open-ended process grounded in the irreducible singularity of lived experience.

This relational configuration becomes especially significant when considering the internalization of inequalities and oppressions that manifest as deeply ingrained biases within the subject’s consciousness. Critical phenomenology allows us to read these structures not as mere individual distortions, but as embodied social configurations that translate into perceptual patterns, affective and corporeal dispositions, and spatial and temporal modes of inhabiting the world. In this sense, the clinical relationship can be conceived as a space of perceptual and intersubjective reactivation.

A vivid example of such embodied fracture is offered by Frantz Fanon, who describes the experience of the racialized body as follows:

“My body was given back to me sprawled out, distorted, recolored, clad in mourning in that white winter day. The Negro is an animal, the Negro is bad, the Negro is mean, the Negro is ugly; look, a Negro; it’s cold, the Negro is shivering, the Negro is shivering because he’s cold, the small boy is trembling because he’s afraid of the Negro, the Negro is trembling with cold, that cold that chills the bones, the lovely little boy is trembling because he thinks the Negro is trembling with rage, the Negro is trembling with rage, the Negro is trembling” (Fanon, 2008, p. 113).

This passage powerfully illustrates what it means to lose the unity of one’s bodily schema – to be returned to the world as a fragmented object within the gaze of the other. The therapeutic relationship can intervene in these perceptual disarticulations by offering a shared space of bodily re-affiliation through a therapeutic Paarung – one that does not normalize the experience, but restructures it by opening new horizons of meaning.

As Husserl pointed out in the Husserl (2002), modern science has progressively lost its grounding in the *Lebenswelt*, absolutizing objectifying models and forgetting the primacy of lived experience. Similarly, clinical practice risks reducing the patient to a bearer of symptoms, thereby neglecting the situated, embodied, and relational dimension of subjectivity. Diagnostic judgment, in this light, may become a tool of naturalizing suffering rather than a means of understanding and transformation.

It is within this fracture that Paarung reveals its ethical-epistemological significance. It is not only the condition of possibility for perceiving the other but also the relational structure that can guide the formation of new, shared judgments – judgments liberated from the constraints of prejudice. Phenomenologically understood, judgment is not the application of a pre-established norm, but the result of a co-constitution of meaning grounded in reciprocal responsibility and attunement to situated experience.

The therapeutic encounter thus becomes a space for interrupting the passive associations that fuel perceptual alienation, but also – and more importantly – a generative site in which a new orientation of the self may emerge. In this framework, Paarung becomes the principle that can guide a therapeutic process aimed at restoring the spatial and temporal continuity of subjective experience, working not only on the content of the narratives but also on their perceptual and relational structure.

This approach fruitfully resonates with Ludwig Binswanger’s conception of the therapeutic relationship as a *We-ness* (Wirheit), that is, an originary relationality between clinician and patient. This dimension does not represent a mere aggregation of subjectivities, but rather a transcendental condition for the very possibility of intersubjectivity and, consequently, of any phenomenological manifestation of spatiality and temporality. We-ness thus prefigures a shared space that is not derivative but constitutive of care, and it finds its deepest structural correlate in Paarung.

In this sense, the context of care is not a neutral background for the clinical encounter, but the result of a shared choice: who determines the context? Who sets its boundaries and meanings? The phenomenological response to these questions is not based on *a priori* categories, but on lived experience as the only reality from which a genuine reorganization of the self’s spatial and temporal orientation can emerge.

Understood in this way, the clinical relationship becomes a process of relational individuation (*in-related self*), in which identity is not constituted through similarity or assimilation, but as a differential unity capable of holding together distance and proximity, alterity and identity. It is within this space – fragile yet generative – that the subject may rediscover the possibility of inhabiting their own body, time, and world.
